# CFTR mediates noradrenaline-induced ATP efflux from DRG neurons

**DOI:** 10.1186/1744-8069-7-72

**Published:** 2011-09-24

**Authors:** Takeshi Kanno, Tomoyuki Nishizaki

**Affiliations:** 1 Division of Bioinformation, Department of Physiology, Hyogo College of Medicine 1-1 Mukogawa-cho, Nishinomiya, 663-8501, Japan

**Keywords:** Noradrenaline, Dorsal root ganglion, neuron, ATP efflux, cystic fibrosis transmembrane conductance regulator

## Abstract

In our earlier study, noradrenaline (NA) stimulated ATP release from dorsal root ganglion (DRG) neurons as mediated via β_3 _adrenoceptors linked to G_s _protein involving protein kinase A (PKA) activation, to cause allodynia. The present study was conducted to understand how ATP is released from DRG neurons. In an outside-out patch-clamp configuration from acutely dissociated rat DRG neurons, single-channel currents, sensitive to the P2X receptor inhibitor PPADS, were evoked by approaching the patch-electrode tip close to a neuron, indicating that ATP is released from DRG neurons, to activate P2X receptor. NA increased the frequency of the single-channel events, but such NA effect was not found for DRG neurons transfected with the siRNA to silence the cystic fibrosis transmembrane conductance regulator (CFTR) gene. In the immunocytochemical study using acutely dissociated rat DRG cells, CFTR was expressed in neurons alone, but not satellite cells, fibroblasts, or Schwann cells. It is concluded from these results that CFTR mediates NA-induced ATP efflux from DRG neurons as an ATP channel.

## Findings

The dorsal root ganglion (DRG) is a relay point in pain tracks and a variety of receptors expressed in DRG neurons engage transmission and modulation of pain. ATP receptors, that include ionotropic P2X receptors and metabotropic P2Y receptors, serve as a pain mediator in the DRG [1-9]. A growing body of evidence has pointed to the role of P2X receptors in acute, inflammatory and neuropathic pain, P2X_3 _receptor in spontaneous pain and heat hyperalgesia, P2X_2/3 _receptor in acute mechanical allodynia, and P2Y_2 _receptor in transient receptor potential vanilloid receptor 1-mediated thermal hypersensitivity.

Noradrenaline (NA)/adrenoceptor also participates in the regulation of pain in the DRG. Adrenoceptors are divided into α_1 _receptors that include α_1a_, α_1b _and α_1d _subtypes, α_2 _receptors that include α_2a_, α_2b_, α_2c _and α_2d _subtypes, and β receptors that include β_1_, β_2_, β_3 _and β_4 _subtypes. Peripheral nerve injury causes sympathetic nerve sprouting into the DRG [10,11]. Peripheral nerve axotomy induces hyperexcitation of DRG neurons via a sympathetic pathway, responsible for neuropathic pain such as hyperalgesia and allodynia [12]. Sciatic nerve injury upregulates expression of mRNAs for α_1b_, α_2a_, α_2b_, and β_2 _adrenoceptors in DRG neurons, and NA potentiates slow type of ATP-evoked currents via α_1 _adrenoceptors, that are monitored from DRG neurons [13]. Notably, NA stimulates ATP release from DRG neurons as mediated via β_3 _adrenoceptors linked to G_s _protein involving protein kinase A (PKA) activation, to cause allodynia [14]. DRG neurons form no synapse in the DRG, and therefore, ATP, to activate the receptors, is not released from presynaptic terminals. Then, the big question addressing is where ATP comes from in the DRG. The present study aimed at answering this question.

**Figure 1:**
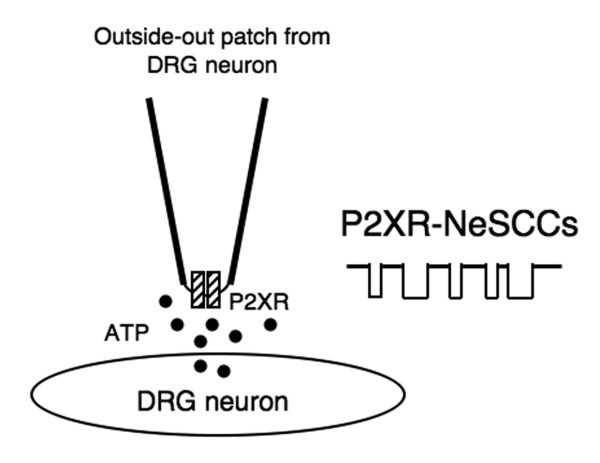
**Schematic diagram for P2XR-NeSCC recording**. Outside-out patches were made from acutely dissociated rat DRG neurons. When the patch-electrode tip is brought within 5 μm close to a neuron, P2XR-NeSCCs are evoked in response to ATP released from DRG neurons.

We utilized an outside-out patch-clamp as a biosensor to monitor ATP mobilizations [14]. Briefly, outside-out patches were made from acutely dissociated rat DRG neurons, and single-channel currents, sensitive to pyridoxal phosphate-6-azophenyl-2',4'-disulfonic acid (PPADS), an inhibitor of P2X receptors, were recorded by approaching the patch-electrode tip close to a DRG neuron. If ATP is released from a neuron, then P2X receptor in an outside-out patch should be activated, to generate single-channel currents ['P2X receptor-mediated neuron-evoked single-channel currents' (P2XR-NeSCCs)] (Figure 1).

**Figure 2:**
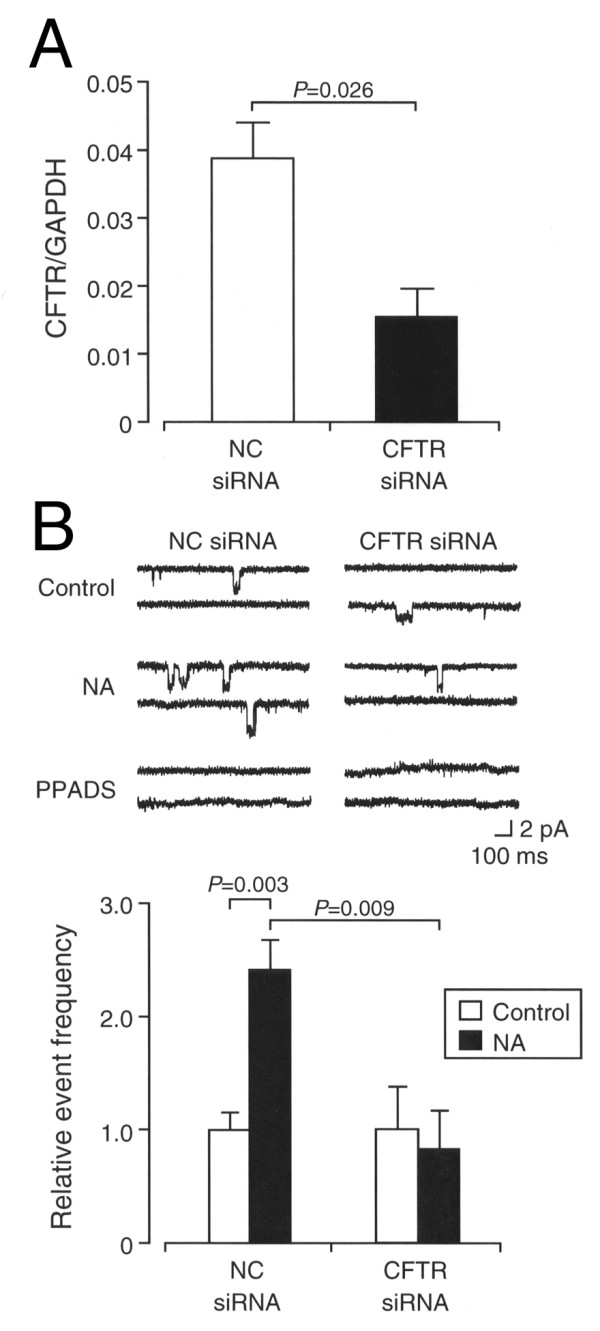
**ATP efflux through CFTR**. Acutely dissociated rat DRG neurons were transfected with the NC siRNA or the CFTR siRNA together with eGFP-expression vector to identify transfected cells. (**A**) Single-cell quantitative real-time RT-PCR. The expression level of the CFTR mRNA was normalized by the expression level of the GAPDH mRNA. In the graph, each value represents the mean (± SEM) normalized CFTR mRNA expression (CFTR mRNA/GAPDH mRNA)(n = 8 independent experiments). *P *value, unpaired *t*-test. (**B**) P2XR-NeSCCs recording. Outside-out patches were made from DRG neurons without transfection, and NeSCCs were monitored before and after treatment with NA (100 μM) in the presence and absence of PPADS (10 μM) by approaching the patch-electrode tip near to neurons transfected with the NC siRNA or the CFTR siRNA. Typical P2XR-NeSCCs are shown in the upper column. The holding potential was -70 mV. In the graph, the frequency of P2XR-NeSCCs was normalized by regarding the frequency before application with NA (Control) as 1 and each value represents the mean (± SEM) normalized event frequency (n = 6 independent patches). *P *values, Dunnett's test.

In our earlier study, NA increased the frequency of P2XR-NeSCCs, i.e., NA stimulated ATP release from DRG neurons [14]. The NA effect was abolished by CFTRinh-172, a selective inhibitor of cystic fibrosis transmembrane conductance regulator (CFTR), while it was not affected by latrunculin B, an inhibitor of vesicular transport, or botulinum toxin A, an inhibitor of vesicular exocytosis [14]. This raises the possibility that NA promotes ATP efflux from DRG neurons through CFTR but not vesicular ATP release. To obtain direct evidence for this, we constructed the siRNA to silence the CFTR-targeted gene (CFTR siRNA). In the single-cell quantitative real-time reverse transcription-polymerase chain reaction (RT-PCR), expression of the CFTR mRNAs was significantly suppressed for acutely dissociated rat DRG neurons transfected with the CFTR siRNA (Figure 2A). In the single channel recording, NeSCCs, that are blocked by PPADS (10 μM), i.e., P2XR-NeSCCs, were obtained by bringing the patch-electrode tip near to neurons transfected with the negative-control siRNA (NC siRNA) or the CFTR siRNA (Figure 2B). When neurons transfected with the NC siRNA were treated with NA (100 μM), the frequency of P2XR-NeSCCs was significantly increased as compared with the frequency before treatment (Figure 2B). In contrast, NA-induced increase in the frequency of P2XR-NeSCCs was not obtained for neurons with knocking-down CFTR (Figure 2B). Taken together, these results indicate that NA-stimulated ATP efflux from DRG neurons is CFTR dependent.

**Figure 3:**
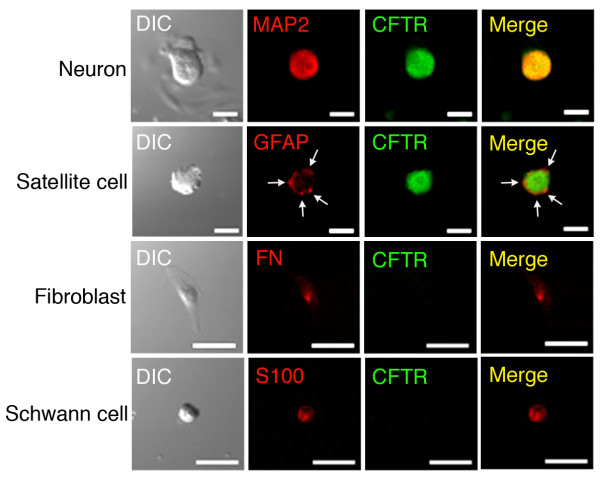
**Expression of CFTR in DRG cells**. Acutely dissociated rat DRG cells were immunostained with antibodies against MAP2, GFAP, fibronectin (FN), or S100 together with an anti-CFTR antibody. DIC, differential interference contrast. Bars, 50 μm. Note that satellite cells around a neuron are detected with an anti-GFAP antibody (arrows) and that immunoreactive signals against an anti-CFTR antibody are found with DRG neurons only, but not satellite cells, fibroblast, or Schwann cell.

To see expression of CFTR in DRG cells, we carried out immunocytochemical study using acutely dissociated rat DRG cells. DRG neurons, fibroblasts, and Schwann cells were identified with antibodies against microtubule-associated protein 2 (MAP2), fibronectin (FN), and S-100, respectively (Figure 3). Satellite cells surrounding neurons were detected with an antibody against glial fibrillary acidic protein (GFAP)(Figure 3). Immunoreactive signals against an anti-CFTR antibody were homogenously found with whole cell bodies of neurons alone (Figure 3). In contrast, satellite cells, fibroblasts, or Schwann cells exhibited no signal against an anti-CFTR antibody (Figure 3). This confirms that CFTR is actually expressed in DRG neurons.

CFTR is an ABC cassette protein and functions as Cl^- ^and ATP channels [15,16]. Intriguingly, CFTR is activated by PKA phosphorylation of the regulatory domain [17,18]. In our earlier study, NA-induced increase in the frequency of P2XR-NeSCCs was inhibited by the selective β_3 _adrenoceptor inhibitor SR59230A, knocking-down β_3 _adrenoceptors, or the PKA inhibitor H-89 [14], indicating the implication of β_3 _adrenoceptors linked to G_s _protein involving PKA activation in the ATP efflux. Overall, NA appears to promote ATP efflux from DRG neurons in a CFTR-dependent manner as mediated via a β_3 _adrenoceptor/PKA signaling pathway.

In summary, the results of the present study provide evidence that CFTR mediates NA-induced ATP efflux from DRG neurons. This may establish a novel pathway for activation of ATP receptors under the control of NA/β_3 _adrenoceptor.

## Materials and methods

### Animal care

All procedures have been approved by the Animal Care and Use Committee at Hyogo College of Medicine and were in compliance with the National Institutes of Health Guide for the Care and Use of Laboratory Animals.

### Preparation of DRG cells

The DRGs in the L4 to L6 segment were isolated from Wistar rats (male, 6 w) and incubated 0.3% (w/v) collagenase at 37°C for 30 min. Then, cells were mechanically dissociated and plated on poly-L-lysine-coated glass cover slips. Experiments were carried out within 12-24 h after preparation.

### Single-channel current recording

Outside-out patches were made from acutely dissociated DRG neurons. Single-channel currents were monitored with an Axopatch-200A amplifier (Axon Instruments Inc., Sunnyvale, CA, USA) in a recording chamber continuously superfused with an extracellular solution [in mM; 135 NaCl, 5 KCl, 2.5 CaCl_2_, 1 MgCl_2_, 5 *N*-2-hydroxyethyl piperazine-*N'*-2-ethansulfonic acid (HEPES), and 10 glucose, pH 7.3] containing 6,7-dinitroquinoxaline-2,3-dione (DNQX)(20 μM) and DL-2-amino-5-phosphonopentanoic acid (DL-AP5)(100 μM) at a flow rate of 2 ml/min at 21-22°C. The patch electrode-filling solution was as follows: (in mM) 135 Cs-gluconate, 5 CsCl, 2 MgCl_2_, 5 ethyleneglycol-bis-(β-aminoethyl ether)-*N*,*N*,*N'*,*N'*-tetraacetic acid, 5 HEPES, 5 MgATP, and 1 Na_2_GTP, pH 7.2. Currents were analyzed using a pClamp software (version 9.2, Axon Instruments Inc.). NA was bath-applied with an air-pressure injector (PicoPump PV 820, World Precision Instruments, Stevenage, UK).

### Construction and transfection of siRNA

The CFTR siRNA was: 5'-GCUUAAAGGAAGAGGAUAUTT-3' and 5'-AUAUCCUCUUCCUUUAAGCTT-3' (B-Bridge International, Inc., Sunnyvale, CA, USA). The NC siRNA with the scrambled sequence, and the same GC content and nucleic acid composition was used as a negative control (B-Bridge International Inc.). Acutely dissociated neurons were transfected with the NC siRNA or the CFTR siRNA together with an enhanced green fluorescence protein (eGFP)-expression vector using a Nucleofector kit (Amaxa GmbH, Cologne, Germany). Single-channel recording and single-cell quantitative real-time RT-PCR were carried out 24 h after transfection.

### Single-cell quantitative real-time RT-PCR

An eGFP-positive single DRG neuron was picked up with a glass capillary containing 18 μl lysis buffer [1× First strand buffer, 0.5 mg/ml bovine serum albumin, RNase inhibitor, 0.6% NP40, 0.6% Tween 20, anti-sense primer for CFTR, anti-sense primer for glyceraldehyde-3-phosphate dehydrogenase (GAPDH), dNTP and 1 mg/ml yeast tRNA], incubated at 70°C for 1 min to break the cell membrane. Cell lysate was incubated in the presence of DNase I at 37°C for 30 min to remove genomic DNAs and at 70°C for 10 min to inactivate DNase I. Then, the sample was incubated in a DTT solution containing SuperScript III reverse transcriptase (Invitrogen, Carlsbad, CA, USA) at 55°C for 40 min followed by at 70°C for 15 min. The gene expression was amplified by double PCRs. Initially, PCR was carried out in the reaction solution containing 10× PCR buffer, dNTPs, oligonucleotide, dimethyl sulfoxide [final concentration, 5% (v/v)], and 5 units of Taq polymerase (Fermentas, St. Leon-Roth, Germany)(final volume, 20 μl) with a PCR Thermal Cycler Dice (Takara, Otsu, Japan) programmed as follows: first step, 98°C for 4 min, the ensuring 40 cycles, 98°C for 1 s, 65°C for 15 s, and 72°C for 30 s. Subsequently, real-time PCR was performed using the first PCR products with a SYBR Green realtime PCR Master Mix (Toyobo, Osaka, Japan) and the Applied Biosystems 7900 real-time PCR detection system (ABI, Foster City, CA, USA) programmed as follows: first step, 98°C for 4 min; the ensuring 40 cycles, 98°C for 1 s, 65°C for 15 s, and 72°C for 30 s. The expression level of rat CFTR mRNA was normalized by that of GAPDH mRNA. Primers used for RT-PCR were as follows: 5'-AATATCCTTAGCCCCTCGGA-3' and 5'-TGGTGGAAACAATGGCACTA-3' for CFTR (accession number M89906) and 5'-CCTTCCGTGTTCCTACCCCCAAT-3' and 5'-CCTCTCTCTTGCTCTCAGTATCCTTGCT-3' for GAPDH (accession number BC059110).

### Immunocytochemistry

Acutely dissociated DRG cells were fixed with 4% (w/v) paraformaldehyde, permeabilized with 0.3% (v/v) Triton X-100, and blocked with 10% (v/v) goat serum in phosphate buffered saline (PBS) at room temperature. Cells were reacted with a mouse monoclonal antibody for MAP2 (1:500), GFAP (1:400), S-100 (1:500) or fibronectin (1:500)(Chemicon, Temecula, CA, USA) and a rabbit polyclonal antibody for CFTR (1:200)(Chemicon) overnight at 4°C followed by a goat anti-rabbit IgG conjugated with Alexa 488 (Molecular Probes, Eugene, OR, USA) and a goat anti-mouse IgG conjugated with Alexa 633 (Molecular Probes) for 60 min at room temperature. Fluorescence-labeled cells were visualized with a confocal scanning laser microscope (Axiovert/LSM510; Carl Zeiss, Oberkochen, Germany).

## Competing interests

The authors declare that they have no competing interests.

## Authors' contributions

All authors read and approved the final manuscript. TN. conducted the majority of the experiments and data and wrote the manuscript. TK contributed to all the experiments and data analysis.
